# Transcriptional Signature of an Altered Purine Metabolism in the Skeletal Muscle of a Huntington's Disease Mouse Model

**DOI:** 10.3389/fphys.2017.00127

**Published:** 2017-03-02

**Authors:** Michal Mielcarek, Ryszard T. Smolenski, Mark Isalan

**Affiliations:** ^1^Department of Life Sciences, Imperial College LondonLondon, UK; ^2^Department of Epidemiology of Rare Diseases and Neuroepidemiology, Poznan University of Medical SciencesPoznan, Poland; ^3^Department of Biochemistry, Medical University of GdańskGdańsk, Poland

**Keywords:** Huntington's disease, skeletal muscle atrophy, purine metabolism, transcriptional deregulation, biomarkers, mouse models

## Abstract

Huntington's disease (HD) is a fatal neurodegenerative disorder, caused by a polyglutamine expansion in the huntingtin protein (HTT). HD has a peripheral component to its pathology: skeletal muscles are severely affected, leading to atrophy, and malfunction in both pre-clinical and clinical settings. We previously used two symptomatic HD mouse models to demonstrate the impairment of the contractile characteristics of the hind limb muscles, which was accompanied by a significant loss of function of motor units. The mice displayed a significant reduction in muscle force, likely because of deteriorations in energy metabolism, decreased oxidation, and altered purine metabolism. There is growing evidence suggesting that HD-related skeletal muscle malfunction might be partially or completely independent of CNS degeneration. The pathology might arise from mutant HTT within muscle (loss or gain of function). Hence, it is vital to identify novel peripheral biomarkers that will reflect HD skeletal muscle atrophy. These will be important for upcoming clinical trials that may target HD peripherally. In order to identify potential biomarkers that might reflect muscle metabolic changes, we used qPCR to validate key gene transcripts in different skeletal muscle types. Consequently, we report a number of transcript alterations that are linked to HD muscle pathology.

## Introduction

Skeletal muscle loss and dysfunction are often associated with aging but are also found in many chronic diseases with diverse etiologies (Vinciguerra et al., 2010). The list includes Huntington's disease (HD), which is a monogenic neurodegenerative disorder caused by a CAG-repeat expansion within the huntingtin gene (*HTT*). The expansion mutation is found in Exon-1 and translates into a polyglutamine stretch (polyQ) in HTT protein, which alters function (for a recent review see Zielonka et al., 2015). To date, the best functional description of HTT is as a “scaffolding protein.” This is because HTT has many interaction partners and these are involved in a wide variety of cellular processes, including gene transcription, intracellular signaling, trafficking, endocytosis, and metabolism (Harjes and Wanker, 2003), hence one might describe HD as a multi-system disorder (Mielcarek, 2015).

In humans, HD is characterized by motor dysfunction, cognitive decline, and a progressive dementia, with the first symptoms typically occurring in midlife (for review see Zielonka et al., 2015). Although HD is still being described as a neurological disorder, there is incontrovertible and growing evidence that peripheral pathology participates in disease progression (Mielcarek, 2015). This is in line with the fact that HTT is normally expressed at high levels in a wide variety of mammalian tissues (Li et al., 1993). Furthermore, pathological high molecular weight HTT aggregates have been identified in many non-central nervous system tissues including skeletal muscle (Moffitt et al., 2009), although interestingly not in hearts (Mielcarek et al., 2014a). Aggregation of mutant HTT is widely-believed to be one of the most important HD molecular pathology signatures and can be detected at an early pre-symptomatic stage in both pre-clinical and clinical HD settings (Davies et al., 1997). HD-related skeletal muscle malfunction has been widely described in both these settings (reviewed in Zielonka et al., 2014a; Mielcarek and Isalan, 2015).

Skeletal muscle constitutes up to 40% of body mass and, in healthy conditions, its function is orchestrated by a balanced network of intrinsic hypertrophic and atrophic signals that maintain metabolic homeostasis (Arinze, 2005; Ehrenborg and Krook, 2009). Any imbalance in these signals may lead to the impairment of muscle regeneration and, consequently, may cause a detrimental dysfunction of skeletal muscle, as has been observed in many chronic diseases (Bassel-Duby and Olson, 2006; Brooks and Myburgh, 2014). We have recently described physiological and functional changes in the skeletal muscles of two well-established HD mouse models, namely R6/2 and *Hdh*Q150 (Mielcarek et al., 2015). We found that these symptomatic HD mouse models had substantial alterations in energy equilibria in various skeletal muscles. This was accompanied by decreased levels of the phosphocreatine/creatine ratios, as well as lower ADP and AMP levels. Moreover, the total pools of the adenine nucleotides were also consistently depleted. Finally, our previous analysis revealed that the EDL muscle showed a slower glycolytic flux from exogenous glucose, as well as less oxidation of glucose (Mielcarek et al., 2015).

In order to underpin our understanding of these metabolic changes, we performed the current transcriptional signature study. For comparability, we employed the same HD mouse model (R6/2) in which we previously described muscle energy imbalances with alterations of purine nucleotides. Since we previously showed that purine metabolism is significantly altered in pre-clinical HD settings (Mielcarek et al., 2015), our current study exclusively focused on the key transcriptional elements of this process as these might serve as important molecular biomarkers for up-coming pre-clinical and clinical studies in HD.

Finally, it should be noted that HD was initially recognized as neurological disorder; although peripheral pathology was noticed, it was generally believed that the changes in peripheral organs were a consequence of CNS degeneration. As a result, over the past decades, researchers concentrated on identifying HD biomarkers that are exclusively attributed to CNS degeneration. However, a number of recently published studies have indicated that skeletal muscle atrophy might be driven by intrinsic mutant HTT functions in muscle (for review Zielonka et al., 2015). Moreover, HD has started to be recognized as a multi-system disorder (Mielcarek, 2015). Hence, there is an urgent need to identify biomarkers that will reflect peripheral pathological changes including those related to HD-skeletal muscle atrophy.

## Materials and methods

### Mouse maintenance and genotyping

The R6/2 HD mouse line was bred and genotyped as previously described (Mielcarek et al., 2015; Agustín-Pavón et al., 2016). All experimental procedures were conducted under a project license from the Home Office, UK and approved by the Animal Welfare and Ethical Review Body of Imperial College London. Experimental groups included the R6/2 mouse model at 12 weeks of age (*n* = 6) and their C57BL/6J littermates (*n* = 6). All animals had unlimited access to water and breeding chow (Special Diet Services, Witham, UK), and housing conditions and environmental enrichment were as described previously (Mielcarek et al., 2015; Agustín-Pavón et al., 2016).

### RNA extraction and taqman real-time PCR expression analysis

Total RNA from skeletal muscles was extracted with the mini-RNA kit (Qiagen, UK), according to the manufacturer's instructions. The reverse transcription reaction was performed using MMLV superscript reverse transcriptase (Invitrogen, USA) and random hexamers (Sigma, USA), as described in an earlier study (Mielcarek et al., 2011). All Taqman qPCR reactions were performed with a LightCycler® 480 Instrument (Roche), as described previously (Agustín-Pavón et al., 2016). Following Taq-man assays from Thermo Fisher Scientific were used in this study Adsl (Mm_00507759_m1); Adssl1 (Mm_00475814_m1); Gart (Mm_00599836_m1); Ppat (Mm_00549096_m1); Aprt (Mm_04207857_g1); Ak1 (Mm_00445475_m1); Gmpr (Mm_00499393_m1); Impdh2 (Mm_00496156_m1); Ada (Mm_00545720_m1); Adk (Mm_00612772_m1); Nt5e (Mm_00501910_m1); Ampd3 (Mm_00477495_m1); Entpd2 (Mm_00515450_m1); Pnp (Mm_00840006_m1); Xdh (Mm_00442110_m1); Prkaa1 (Mm_01296700_m1); Pdk4 (Mm_01166879_m1); Hk2 (Mm_00443385_m1). mRNA copy number was determined in triplicate for each RNA sample by comparison with the geometric mean of three endogenous housekeeping genes (Primer Design, UK), as described (Mielcarek et al., 2011). Stable housekeeping genes for qPCR profiling of various skeletal muscles for HD mouse models were determined using the Primer Design *geNorm*™ *Housekeeping Gene Selection Mouse Kit with PerfectProbe*™ *software*.

### Statistical analysis

Values were presented as mean ± SEM. Statistical analysis was performed using paired Student *t*-tests (Excel) or One-Way Anova SPSS (IBM). A *p*-value of 0.05 was considered as a significant difference.

## Results

**Figure 1:**
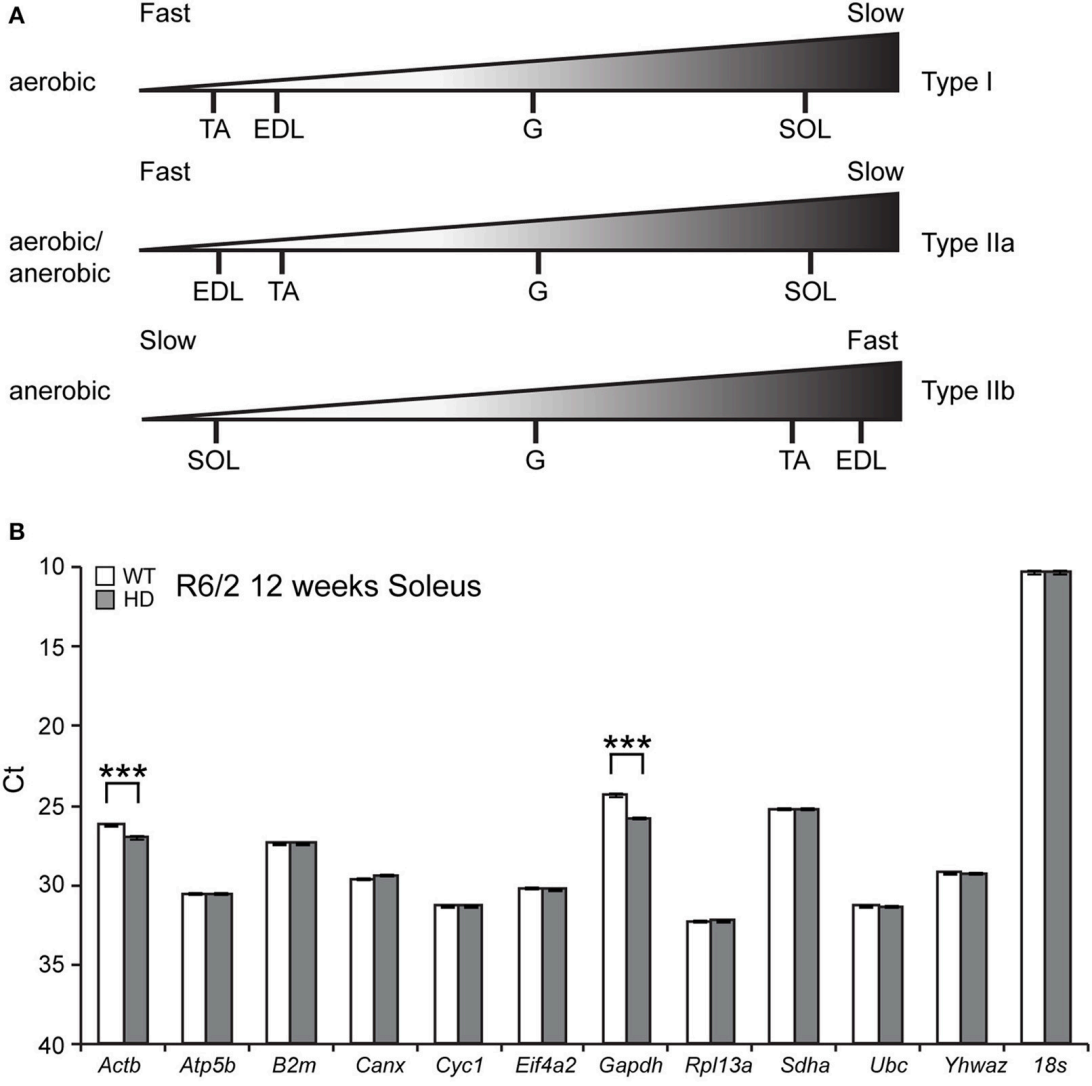
**(A)** Summary of composition of fiber types in the hindlimb skeletal muscles used in this study. **(B)** Identification of reference genes for qPCR from Soleus RNA from the R6/2 mouse model. GeNorm analysis was used to identify optimal reference genes. Raw crossing threshold (Ct) data for a panel of 12 potential reference genes from the geNorm kit in WT and R6/2 mice (12 weeks old) from Soleus. ^***^p < 0.001.

In order to establish the transcriptional signature related to the pathology observed in HD skeletal muscle, we employed the R6/2 transgenic mouse model of HD, representing the fully symptomatic stage of a rapid-onset form of the disease. Since the characteristic energy imbalance was previously detected in all muscle types, we performed a detailed profiling of transcriptional changes related to purine metabolism and energy balance within different muscles. We focused on Extensor Digitus Longum (EDL), Soleus (Sol), Tibialis Anterior (TA) and Gastrocnemius, and Plantaris complex (G/P) because these are composed from metabolically-different muscle fibers. Typically, hindlimb muscles can be divided into two groups (fast and slow) based on the composition of fibers, containing up to four myosin isoforms. Type I fibers are also known as slow twitch fibers and they produce ATP through an aerobic metabolic cycle. Type IIa fibers are also known as intermediate fast oxidative fibers and they produce ATP through both aerobic and anaerobic metabolic pathways. Type IIb fibers are known as fast glycolytic fibers and they produce ATP through an anaerobic metabolic cycle (Schiaffino and Reggiani, 2011). Figure 1A summarizes the hindlimb muscle classification used in this study, based on a previously published study in the C57BL6J mouse strain (Augusto et al., 2004).

Since HD causes large amount of transcriptional deregulation, we performed a systematic study to identify suitable reference genes for use in the expression analysis of different skeletal muscle types. We previously established a number of housekeeping genes that are stable across the different muscle types used in this study, summarized in Table 1 (Mielcarek et al., 2015). We used the geNorm™ Housekeeping Gene Selection Mouse Kit, and associated software, to identify the three most stably-expressed genes in Soleus muscles from R6/2 (Figure 1B). This approach was necessary for our relative quantification method; this uses the geometric mean of three selected reference genes for normalization, to accurately determine gene expression levels in WT and R6/2 skeletal muscle tissue, summarized in Table 1.

**Table 1:** **Summary of housekeeping genes specific for each muscle type in the R6/2 HD mouse model used in this study**.

**Skeletal muscle**	**R6/2 mouse model**
EDL	*B2m, Rpl13a, Yhwaz*
SOL	*B2m, Yhwaz, 18S*
TA	*Atp5b, Rpl13a, Yhwaz*
G/P	*Atp5b, Rpl13a, Yhwaz*

*Genes were selected based on GeNorm analysis to identify optimal reference genes. The following gene transcripts were examined: Atcb, Actin, beta, cytoplasmic, 11461; Gapdh, Glyceraldehydes-3-phosphate dehydrogenase, 14433; Ubc, Ubiquitin C, 22190; B2m, Beta-2-microglobulin, 12010; Ywhaz, Phospholipase A2, 22631; Rpl13a, Ribosomal protein L13a, 22121; Canx, Calnexin, 12330; Cyc1, Cytochrome c-1, 66445; Sdha, Succinate dehydrogenase complex, subunit A, 66945; 18S, 18S rRNA, 19791; Eif4A2, Eukaryotic translation initiation factor 4A2, 13682; Atp5b, ATP synthase subunit, 11947. Some of the genes used in this study have been previously identified as stable reference genes (Mielcarek et al., 2015)*.

Since we showed previously that purine metabolism is altered in HD skeletal muscles (Mielcarek et al., 2015), we next studied the transcriptional signature of genes involved in *de novo* purine synthesis. These were examined in different muscles in the R6/2 HD mouse model, using comparisons to control WT littermates.

**Figure 2:**
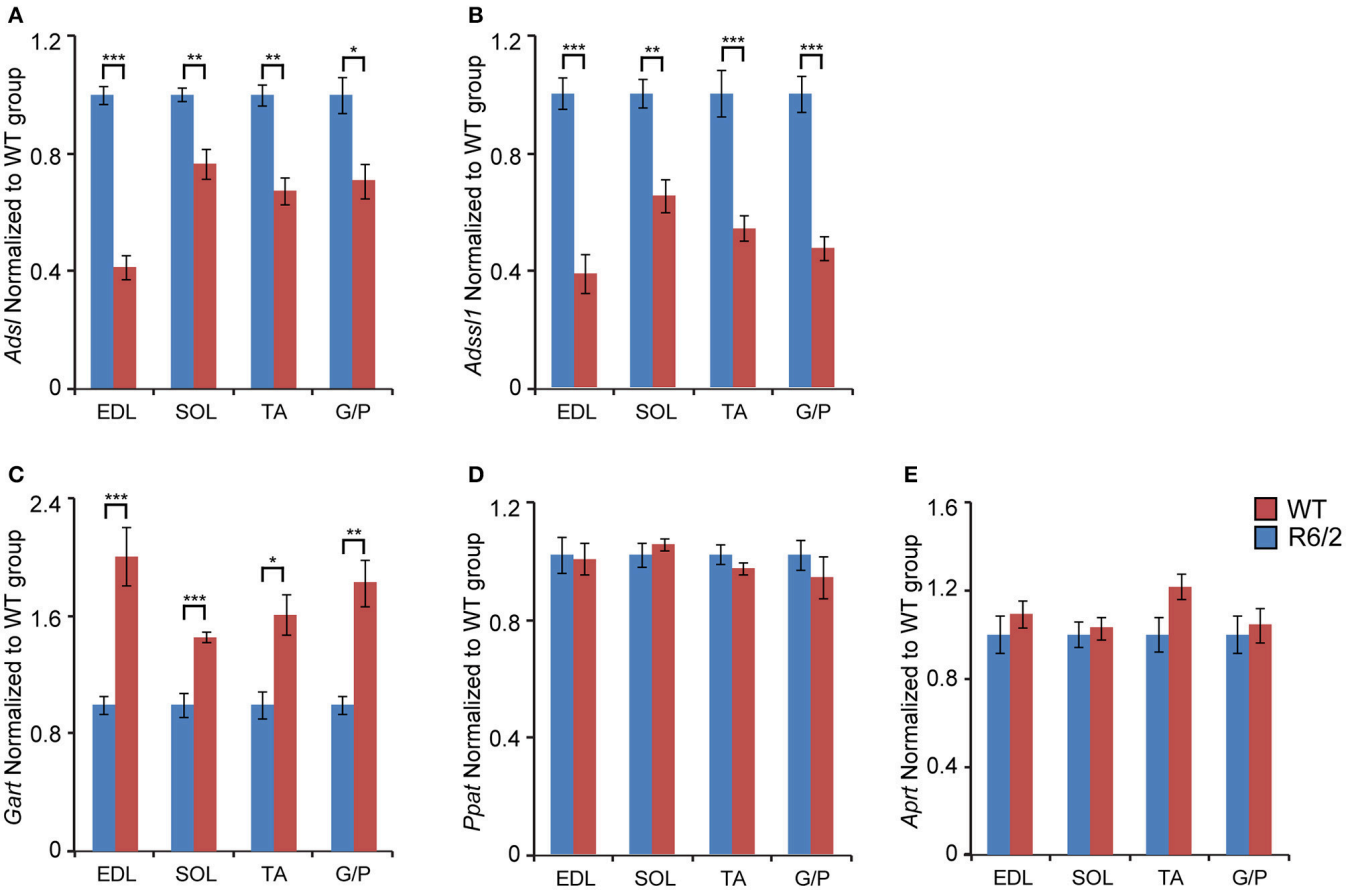
**Transcriptional remodeling of genes involved in *de novo* purine synthesis and salvage pathway**. Transcripts of genes involved in *de novo* purine biosynthesis. **(A)** *Adsl*, Adenylosuccinate lyase; **(B)** *Adssl1*, Adenylosuccinate lyase 1; **(C)** *Gart*, Phosphoribosylglycinamide formyltransferase; **(D)** *Ppat*, Amidophosphoribosyltransferase; and the purine nucleotide salvage pathway **(E)** *Aprt*, Adenine phosphoribosyltransferase; were assessed in different types of skeletal muscle: EDL, Extensor Digitorum Longus; SOL, Soleus; TA, Tibialis Anterior; and G/P, Gastrocnemius and Plantaris complex. All Taqman qPCR-values were normalized to the geometric mean of three housekeeping genes as indicated in the Materials and Methods Section. Error bars are ± SEM (*n* = 6). Student's *t*-test: ^*^p < 0.05, ^**^p < 0.01; ^***^p < 0.001.

First, we found that the transcript levels of two genes involved in the purine nucleotide cycle (PNC) were significantly down-regulated. Adenylosuccinate lyase (*Adsl*, Figure 2A) and Adenylosuccinate lyase 1 (*Adlssl1*, Figure 2B) were reduced by ~40–80% in each type of skeletal muscle.

When examining the mRNA levels of enzymes involved in purine synthesis, we found that Phosphoribosylglycinamide formyltransferase (*Gart*) was significantly up-regulated (up to 2-fold) in each type of skeletal muscle (Figure 2C). In contrast, Adenine phosphoribosyltransferase (*Aprt*) and Amidophosphoribosyltransferase (*Ppat)* mRNA remained unchanged (Figures 2D,E).

**Figure 3:**
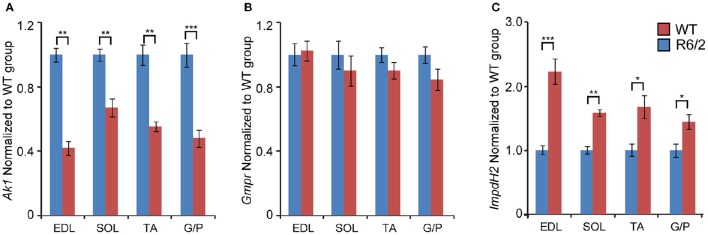
**Transcriptional alteration of genes engaged in conversion of adenine nucleotides**. Transcripts of **(A)** *Ak1, Adenylate kinase 1*; **(B)** *Gmpr, Guanosine monophosphate reductase*; and **(C)** *Impdh2, inosine monophosphate dehydrogenase 2*; were assessed in different types of skeletal muscle: EDL, Extensor Digitorum Longus; SOL, Soleus; TA, Tibialis Anterior; and G/P, Gastrocnemius and Plantaris complex. All Taqman qPCR-values were normalized to the geometric mean of three housekeeping genes as indicated in the Materials and Methods Section. Error bars are ± SEM (*n* = 6). Student's *t*-test: ^*^p < 0.05, ^**^p < 0.01; ^***^p < 0.001.

We also examined the transcriptional changes of genes involved in the conversion of purine nucleotides. We found that mRNA levels of Adenylate kinase 1 (*Ak1*; an enzyme that is involved in the catalysis of the terminal phosphate group between ATP and AMP) were significantly down-regulated in each type of skeletal muscle examined (Figure 3A). Conversely, the transcript levels of Inosine monophosphate dehydrogenase 2 (*Impdh2*), which catalyzes the conversion of IMP (Inosine 5′-phosphate) to XMP (Xanthosine 5′-phosphate), were significantly up-regulated, by up to 2-fold (Figure 3C). The transcript levels of Guanosine monophosphate reductase (*Gmpr*; maintains the intracellular balance of adenine and guanine nucleotides) remained unchanged (Figure 3B).

**Figure 4:**
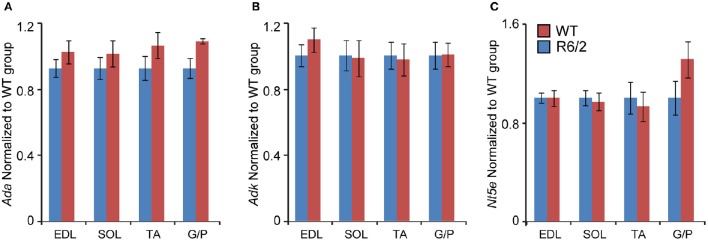
**Unchanged transcriptional levels of genes involved in in adenosine metabolism**. Transcripts of **(A)** *Ada*, Adenosine deaminase; **(B)** *Adk*, Adenosine kinase; and **(C)** *Nt5e*, Ecto-5′-nucleotidase; were assessed in different types of skeletal muscle: EDL, Extensor Digitorum Longus; SOL, Soleus; TA, Tibialis Anterior; and G/P, Gastrocnemius and Plantaris complex. All Taqman qPCR-values were normalized to the geometric mean of three housekeeping genes as indicated in the Materials and Methods Section. Error bars are ± SEM (*n* = 6). Student's *t*-test: *p* > 0.05.

Subsequently, we verified the transcriptional changes of selected genes that are believed to be involved in adenosine metabolism. The mRNA levels of *Ada* (Adenosine deaminase; Figure 4A), *Adk* (Adenosine kinase; Figure 4B) and *Nt5e* (Ecto-5′-nucelotidase; Figure 4C) remained unchanged under all conditions.

**Figure 5:**
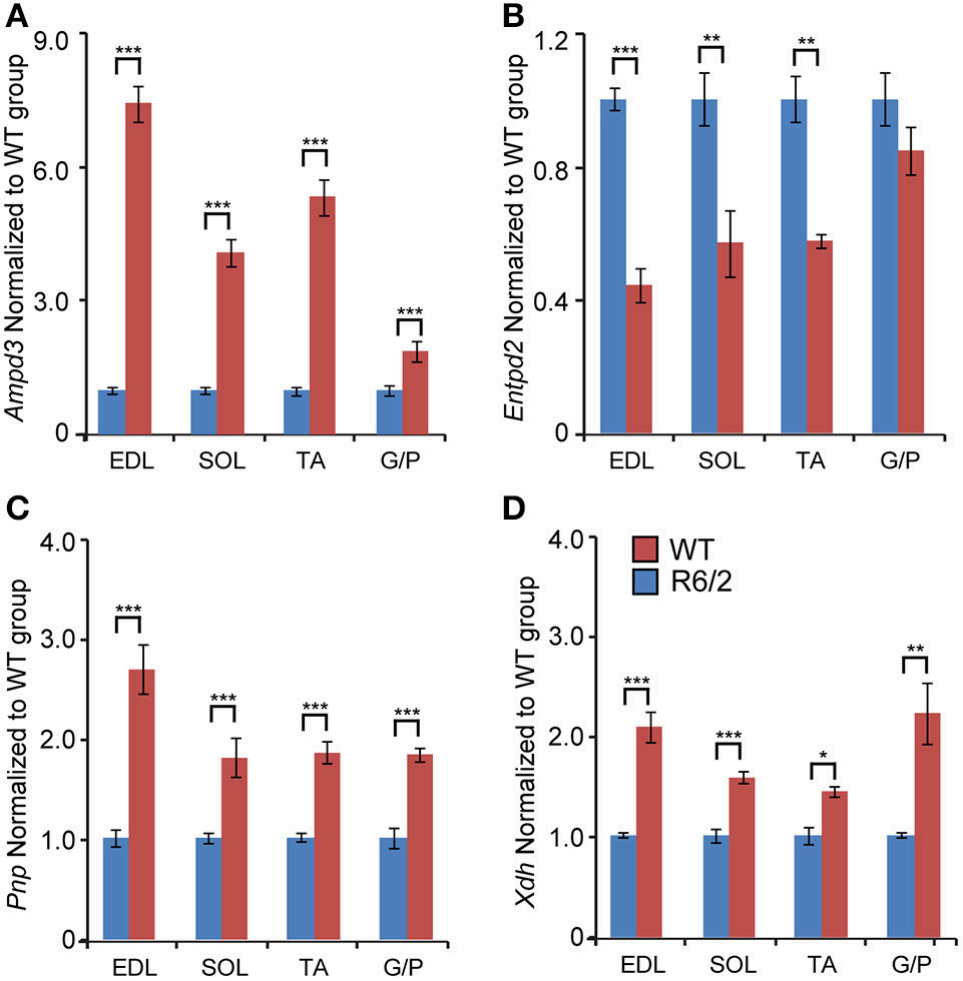
**Transcriptional remodeling of genes involved in purine metabolism and their degradation**. Transcript levels of **(A)** *Ampd3, Adenosine deaminase 3*; **(B)** *Entpd2, Ectonucleoside triphosphate diphosphohydrolase 2*; **(C)** *Pnp, Purine nucleoside phosphorylase*; **(D)** *Xdh, Xanthine dehydrogenase*; assessed in different types of skeletal muscle: EDL, Extensor Digitorum Longus; SOL, Soleus; TA, Tibialis Anterior; and G/P, Gastrocnemius and Plantaris complex. All Taqman qPCR-values were normalized to the geometric mean of three housekeeping genes as indicated in the Materials and Methods Section. Error bars are ± SEM (*n* = 6). Student's *t*-test: ^*^p < 0.05, ^**^p < 0.01; ^***^p < 0.001.

We next assessed the transcriptional signature of genes involved in purine metabolism and their degradation. There was a significant up-regulation of the *Ampd3* (*Adenosine monophosphate deaminase 3*) specifically in the fast-type muscles like EDL (Figure 5A). In fact, the effect was large (up to 9-fold), which is interesting given that *Ampd3* is a marker of skeletal muscle denervation (Fortuin et al., 1996). In addition, we observed a robust *Ampd3* mRNA up-regulation in other muscle types like soleus (up to 5-fold), TA (up to 6-fold), and G/P (up to 2.5-fold).

There was a significant down-regulation across tested conditions of *Entpd2* (Ectonucleoside triphosphate diphosphohydrolase 2; regulates ATP hydrolysis), specifically in fast-type skeletal muscles, such as EDL (Figure 5B). Contrastingly, the mRNA levels of *Pnp* (*Purine nucleoside phosphorylase*) were significantly and uniformly up-regulated across each muscle examined (Figure 5C).

We further validated the expression levels of mRNAs coding for enzymes involved in purine degradation pathways (Yang et al., 2008; Walker et al., 2011). We found that *Xdh* (*Xanthine dehydrogenase*) transcript levels were significantly up-regulated, uniformly across all conditions (Figure 5D).

**Figure 6:**
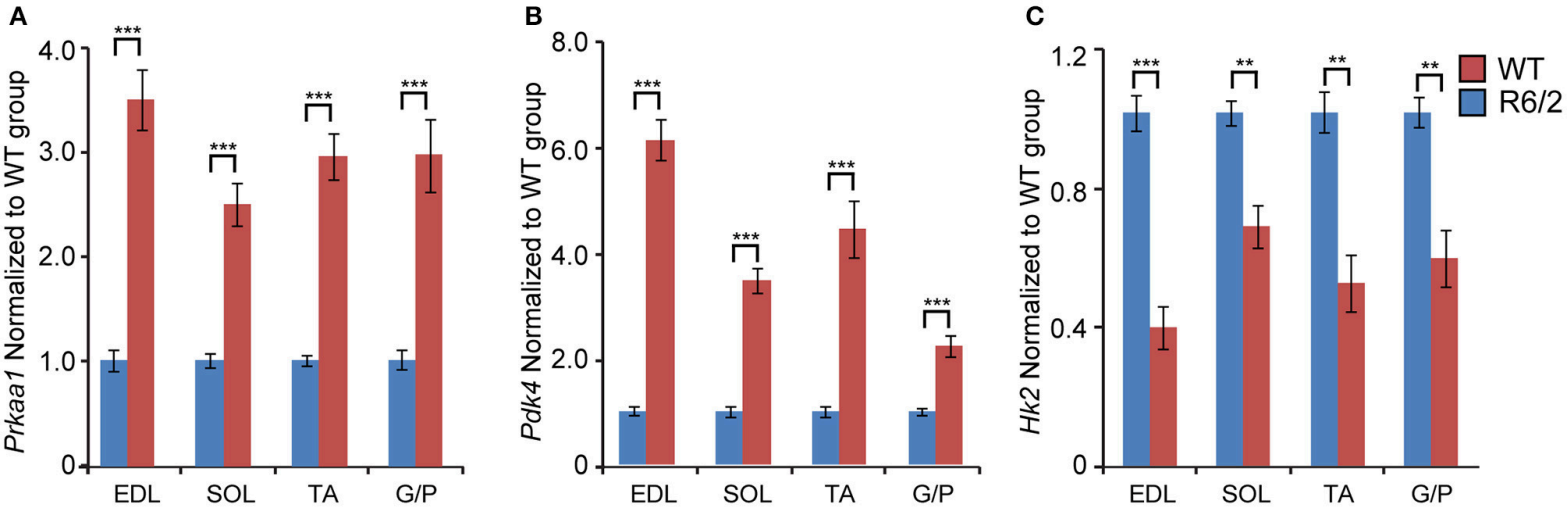
**Transcriptional alteration of genes involved in energy homeostasis**. Transcripts of genes involved in **(A)** *Prkaa1, 5*′*-AMP-activated protein kinase catalytic subunit alpha-1* and **(B)** *Pdk4, Pyruvate dehydrogenase, kinase isozyme 4*; **(C)** *Hk2, Hexokinase 2*; were assessed in different types of skeletal muscle: EDL, Extensor Digitorum Longus; SOL, Soleus; TA, Tibialis Anterior; and G/P, Gastrocnemius and Plantaris complex. All Taqman qPCR-values were normalized to the geometric mean of three housekeeping genes as indicated in the Materials and Methods Section. Error bars are ± SEM (*n* = 6). Student's *t*-test: ^**^p < 0.01; ^***^p < 0.001.

**Figure 7:**
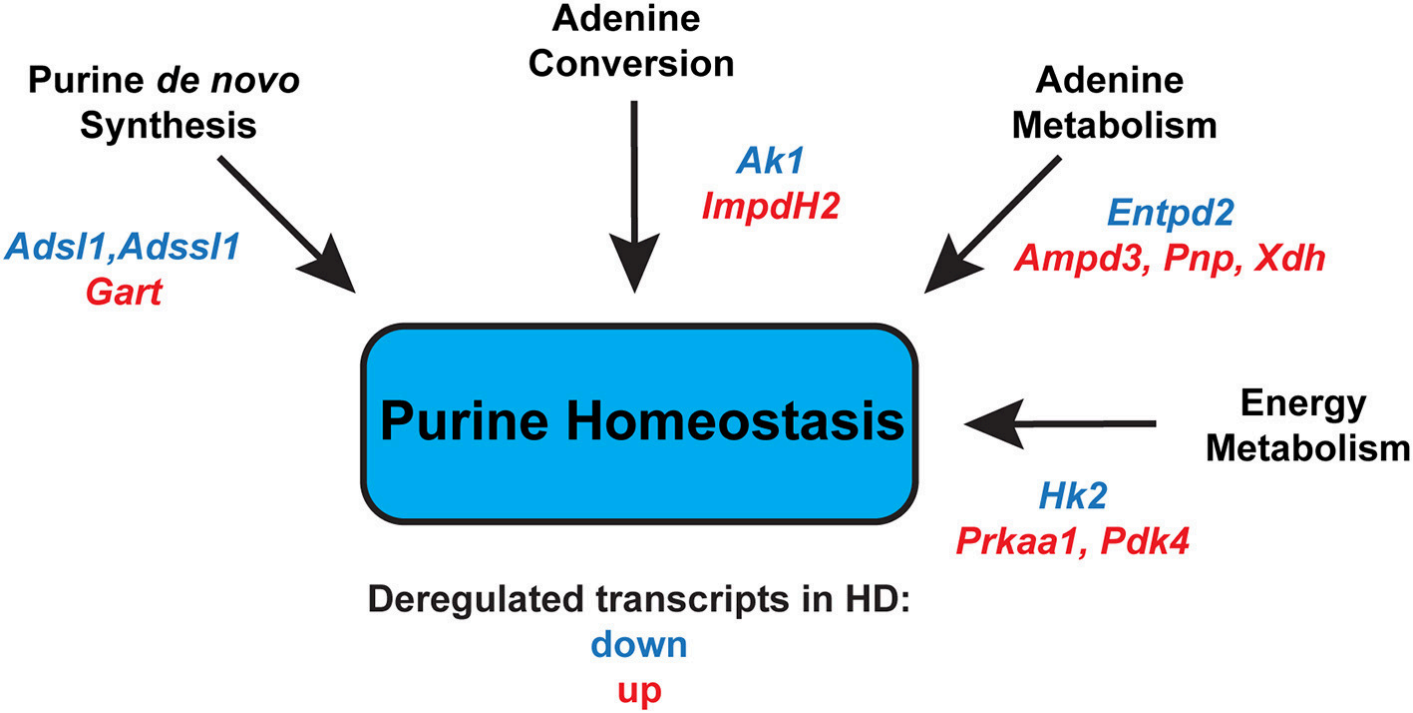
**Summary of significantly deregulated transcripts in HD skeletal muscles that might alter purine homeostasis, leading to an energy imbalance**. *Adsl, Adenylosuccinate lyase*; *Adssl1, Adenylosuccinate lyase 1*; *Gart, Phosphoribosylglycinamide formyltransferase*; *Ak1, Adenylate kinase 1*; *Impdh2, inosine monophosphate dehydrogenase 2*; *Entpd2, Ectonucleoside triphosphate diphosphohydrolase 2*; *Ampd3, Adenosine deaminase 3*; *Pnp, Purine nucleoside phosphorylase*; *Xdh, Xanthine dehydrogenase*; *Hk2, Hexokinase 2*; *Prkaa1*; *5*′*-AMP-activated protein kinase catalytic subunit alpha-1*; *Pdk4, Pyruvate dehydrogenase, kinase isozyme 4*.

Finally, we assessed the expression levels of two genes involved in energy metabolism. *Prkaa1* (*5*′*-AMP-activated protein kinase catalytic subunit alpha-1*) mRNA levels were significantly up-regulated up to 3-fold, in both fast- and slow-type skeletal muscle (Figure 6A). Similarly, *Pdk4* (*Pyruvate dehydrogenase, kinase isozyme 4*) transcript levels were found to be up-regulated up to 6-fold in fast-type muscle (EDL; Figure 6B). The transcript levels of *Hk2* (*Hexokinase 2*) were significantly down-regulated, particularly in the fast-type muscles like EDL (Figure 6C). Thus, one may conclude that there was a significant deregulation of both these energy metabolism genes, whose function we previously reported to be deregulated in HD mouse models (Mielcarek et al., 2015). Overall, the changes in transcript levels in different muscles in the R6/2 HD mouse model are summarized in Figure 7.

## Discussion

HD is an autosomal dominant, progressive, neurodegenerative disorder for which there are currently only symptomatic treatments, reviewed in Zielonka et al. (2015). There is a clear need to develop disease modifying therapies and the progress of these can be informed by new insights into the underlying molecular pathologies. For instance, it is becoming more-and-more apparent that HD can be described as mitochondriopathy: multiple studies have described mitochondrial defects, both *in vitro* and *in vivo*, that might play a central role in HD CNS degeneration (for review see Damiano et al., 2010). Furthermore, it is emerging that HD has a peripheral component contributing to its pathology, including skeletal muscle atrophy (Zielonka et al., 2014a) and heart failure (Zielonka et al., 2014b). In fact, HD-related cardiomyopathy is characterized by brady- and tachyar-rhythmias, variations in heart rates and cardiac remodeling (Mielcarek et al., 2014a), and these are in line with recently published clinical data (Stephen et al., 2015). Consequently, it has been shown that these cardiac pathologies might be caused by significant alterations in mitochondrial structure, including the loss of mitochondrial elongated shapes and diffused mitochondrial densities (Mihm et al., 2007; Kiriazis et al., 2012). Supporting this view of altered mitochondrial energy metabolism, we observed an increased level of purine catabolites in the heart mass of HD mouse models (Toczek et al., 2016a,b). In addition, reactive oxygen species (ROS) play an important role in mitochondrial dysfunction, especially in skeletal muscle atrophy (Zuo et al., 2015; Zuo and Pannell, 2015) and heart failure (He and Zuo, 2015). Similarly, ROS overproduction has been linked to many neurodegenerative diseases (Manoharan et al., 2016), including HD (Ayala-Peña, 2013). In fact, it has been shown that mutant HTT aggregation directly leads to increased ROS production *in vitro* (Hands et al., 2011).

A mitochondrial dysfunction is also present in HD skeletal muscle. For example, HD subjects develop a deficit in mitochondrial oxidative metabolism in skeletal muscles (Saft et al., 2005). This was shown in muscle cell cultures that exhibited abnormalities in mitochondrial membrane potential and cytochrome 3 release (Ciammola et al., 2006). Additionally, our recently published study in HD pre-clinical settings clearly identified an energy deficit and altered metabolism of purine nucleotides, spanning both fast and slow types of skeletal muscle (Mielcarek et al., 2015). This mechanism underpins the progressive impairment of the contractile characteristics of skeletal muscles in HD. In fact, we previously reported that the pool of ATP, ADP, and NAD+ was depleted in fast (EDL) and slow (Soleus) types of skeletal muscle, in R6/2 and *Hdh*Q150 mouse models (Mielcarek et al., 2015). Hence, the aim of this study was to identify a transcriptional signature that is linked to the observed imbalance in purine metabolism and energy production, in order to identify a set of novel biomarkers for HD-related pathology.

A key feature of our study is that we performed our analysis on different types of skeletal muscle, chosen for their contrasting fiber compositions. Therefore, we carried out our transcriptional analysis on EDL (fast-type muscle), Soleus (slow-type muscle), and TA or G/P (mixed fiber compositions). It should be emphasized that skeletal muscle atrophy in HD affects both types of muscles (slow and fast; Sathasivam et al., 1999; Mielcarek et al., 2015). However, the process of atrophy is greater in type II fibers (fast), relative to type I (slow; Ribchester et al., 2004), which in turn may result in a fast-to-slow twitch conversion.

Muscle atrophy can also alter gene expression and *Ampd3* is believed to be a marker of muscle disuse and muscle denervation (Fortuin et al., 1996). Interestingly, we found a robust up-regulation of *Ampd3* transcript levels (up to 9-fold). This is in line with our previous data, indicating that HD muscles are becoming denervated due to loss of motor units function or their inactivity (Mielcarek et al., 2015).

Moreover, we found changes in gene expression that tie in to our previous observations of altered metabolism in HD skeletal muscle (Mielcarek et al., 2015). For example, there was a significant up-regulation (up to 3-fold) of genes such as *Pnp* and *Xdm*, which are linked directly to purine metabolism. We also noticed a significant up-regulation of transcripts that are involved in energy homeostasis, including *Prkaa1* (up to 4-fold) and *Pdk4* (up to 6-fold; for a review see Ehrenborg and Krook, 2009). On the other hand, we found a number of gene transcripts to be down-regulated and these are linked to *de novo* purine synthesis, like *Adsl* and *Adssl1*, followed by *Entpd2* and *Hk2* (both involved in purine metabolism).

In summary, we report the first transcriptional signature that represents changes in energy homeostasis and an altered purine metabolism in the skeletal muscles of an HD mouse model (Figure 7). We believe that the genes used here might provide a useful set of HD biomarkers, both for tracking disease progression and for developing therapies. In fact, there is a growing interest in including skeletal muscle malfunction as a therapeutic target to delay disease progression in HD (Mielcarek et al., 2014b; Zielonka et al., 2014a; Mielcarek, 2015). For example, it was recently shown that reversing skeletal muscle malfunction, by blocking the myostatin pathway, extended the lifespan of the R6/2 mouse model (Mielcarek et al., 2014b). Additionally, HD skeletal muscle shows a denervation-like phenotype, which might be linked to the deregulated HDAC4-myogenin axis (Mielcarek et al., 2015), and HDAC4 is a valid therapeutic target in HD (Mielcarek et al., 2011, 2013).

Ultimately, lowering levels of mutant Huntingtin in HD settings is the most widely accepted therapeutic strategy and here it would be useful to have a set of biomarkers for both central and peripheral (e.g., skeletal muscle) pathology. In this context, we recently published an approach based on artificial zinc finger transcription repressors, targeted to mutant HTT (Garriga-Canut et al., 2012; Agustín-Pavón et al., 2016). We showed that a single AAV delivery treatment can repress the mutant gene in mouse brains for up to 6 months (Agustín-Pavón et al., 2016) and the treatment has the potential to be extended to HD muscle therapy. Hence, our panel of newly identified HD-associated transcripts, which are related to purine metabolism and energy imbalance, might serve as a biomarker platform in such pre-clinical settings. Finally, since HD-related skeletal muscle atrophy shares many molecular and physiological mechanisms with muscle cachexia in cancer mouse models (Mielcarek and Isalan, 2015), it is likely that our set of transcriptional biomarkers may have even wider applications in these settings.

## Author contributions

Conceived and designed the experiments: MM, RS, MI. Performed the experiments: MM. Analyzed the data: MM, RS, MI. Wrote the manuscript: MM, MI.

### Conflict of interest statement

The authors declare that the research was conducted in the absence of any commercial or financial relationships that could be construed as a potential conflict of interest.
